# Supplementary material for: Identification of photoperiod-induced specific miRNAs in the adrenal glands of Sunite sheep (Ovis aries)
Source: Front Vet Sci. 2022 Jul 22;9:888207. doi: 10.3389/fvets.2022.888207 (PMC9354845; doi:10.3389/fvets.2022.888207)
Supplement: Supplementary file 1 [file Data_Sheet_1.ZIP › Supplementary file/miRDeep2 file.zip!/miRDeep2 file/Know_Structure/SP42A3/oar-mir-379.pdf]

miRBase precursor : oar-mir-379
Total read count : 21434
oar-miR-379-5p read count : 20032
oar-miR-379-3p read count : 1397
remaining reads : 5

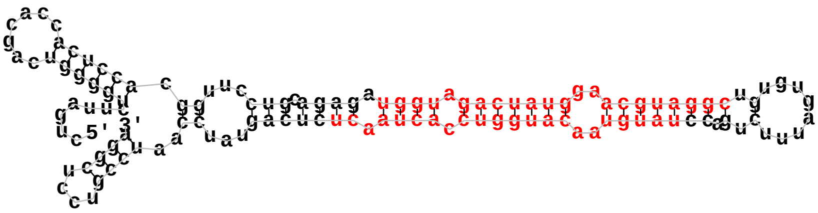

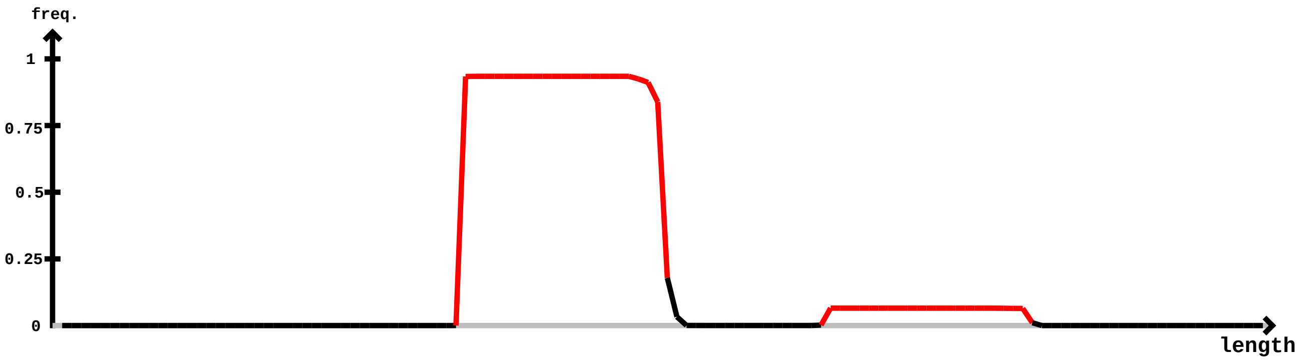


```
                                                                                  oar-miR-379-3p
                                              oar-miR-379-5p
5'- cugauucuggggucagcaccacuccacgguuccugcagagaugguagacuauggaacguaggcugugugauuucugaccuauguaacaugguccacuaacucucaguauccaauccguccucgga  -3'   exp
    .......((((((.......)))))).((...(((.((((.((((.(((((((..((((((((.(........).).)))))))..))))))).)))).)))))))...))..((((....))))   reads      mm
    ........................................augguagacuauggaacguag................................................................   1          0
    ........................................augguagacuauggaacguagg...............................................................   4          0
    .........................................ugguagacuauggaacg...................................................................   1          0
    .........................................ugguagacuauggaacgu..................................................................   214        0
    .........................................ugguagacuauggaacgua.................................................................   279        0
    .........................................ugguagacuauggaacguag................................................................   1561       0
    .........................................ugguagacuauggaacguagg...............................................................   14131      0
    .........................................ugguagacuauggaacguaggc..............................................................   3125       0
    .........................................ugguagacuauggaacguaggcu.............................................................   698        0
    .........................................ugguagacuauggaacguaggcug............................................................   1          0
    .........................................ugguagacuauggaacguaggcugu...........................................................   2          0
    ..........................................gguagacuauggaacguagg...............................................................   14         0
    ..........................................gguagacuauggaacguaggc..............................................................   1          0
    ..........................................................uaggcugugugauuucugaccu.............................................   1          0
    ..............................................................cugugugauuucugacU..............................................   1          1
    ..............................................................cugugugauuucugacc..............................................   1          0
    ..............................................................Gugugugauuucugacc..............................................   1          1
    ..............................................................cugugugauuucugaccuauguaacaugguccacuaac.........................   1          0
    ..............................................................................cuauguaacaugguccacu............................   1          0
    ..............................................................................cuauguaacaugguccacua...........................   1          0
    ..............................................................................cuauguaacaugguccacuaac.........................   30         0
    ..............................................................................cuauguaacaugguccacuaacu........................   2          0
    ...............................................................................uauguaacaugguccacC............................   1          1
    ...............................................................................uauguaacaugguccacu............................   6          0
    ...............................................................................uauguaacaugguccacua...........................   10         0
    ...............................................................................uauguaacaugguccacuaa..........................   4          0
    ...............................................................................uauguaacaugguccacuaac.........................   1116       0
    ...............................................................................uauguaacaugguccacuaacu........................   226        0
```
